# Acceleration of pancreatic tumorigenesis under immunosuppressive microenvironment induced by Reg3g overexpression

**DOI:** 10.1038/cddis.2017.424

**Published:** 2017-09-07

**Authors:** Xiulan Liu, Zhongshi Zhou, Qi Cheng, Hongjie Wang, Hui Cao, Qianqian Xu, Yali Tuo, Li Jiang, You Zou, Hongyu Ren, Ming Xiang

**Affiliations:** 1 Department of Pharmacology, School of Pharmacy, Tongji Medical College, Huazhong University of Science and Technology, Wuhan 430000, China; 2 Section of Neurobiology, Torrey Pines Institute for Molecular Studies, Port Saint Lucie, FL, USA; 3 Department of Biliary and Pancreatic Surgery, Affiliated Tongji Hospital, Tongji Medical College, Huazhong University of Science and Technology, Wuhan 430000, China; 4 Department of Gastrointestinal Surgery, Affiliated Tongji Hospital, Tongji Medical College, Huazhong University of Science and Technology, Wuhan 430000, China; 5 Department of Digestive Disease, Affiliated Xiehe Hospital, Tongji Medical College, Huazhong University of Science and Technology, Wuhan 430022, China

## Abstract

Reg3g is a potential risk for pancreatic ductal adenocarcinoma (PDAC). We previously demonstrated that Reg3g promoted pancreatic carcinogenesis via a STAT3 signaling pathway in a murine model of chronic pancreatitis. Whether the immune response is involved in tumorigenesis induced by Reg3g remains unknown. In this study, Reg3g-regulated tumor immunity was evaluated in tumor-implanted murine models, immune cells, and tumor microenvironment. In mice that had been orthotopically or ectopically implanted with Panc02 cells, Reg3g overexpression increased EGFR and Ki67, diminished MHC-I and caspase-3 expression, and accelerated growth of tumors. By interacting with PD-1/PD-L1, Reg3g also promoted differentiation of Tregs and recruitment of MDSC, retarded maturation of DCs and inactivation of CD8^+^ T cells, and suppressed cross-priming of CD8^+^ T-cell responses by DCs in tumor-bearing mice. Knockdown of Reg3g delayed tumor development in normal mice, but not in CD8^+^ T-cell-deficient mice. *In vitro*, Reg3g upregulated EGFR in DCs, activated heme oxygenase-1 (Hmox1) involved JAK2/STAT3 signaling, raised levels of Th2 cytokines in and suppressed maturation of DCs, and enhanced tumor cell proliferation. These results reveal a novel role of Reg3g as an immunosuppressive promoter that weakens tumor-specific antigenicity and suppresses antitumor effects of CD8^+^ T cells in a murine model of pancreatic cancer. Reg3g produces these effects by activating the JAK2/STAT3 signaling pathway in DCs, triggering the generation of an immunosuppressive tumor microenvironment.

Pancreatic ductal adenocarcinoma (PDAC) is a malignant tumor of the exocrine pancreas. It is most often diagnosed in the later stages with metastasis. During the initiation and development of PDAC, immunosuppressive T cells, tumor-infiltrating Tregs^1^ and cytokines^2^ accumulate in tumor tissue, forming a unique tumor microenvironment (TME), which suppresses activation of both innate and adaptive elements of immune surveillance.^3^ In particular, DCs, the professional antigen-presenting cells, have a central role in balancing tumor immune responses between tolerance induction and immune activation.^4^ Specifically, the tumor cells induce the secretion of cytokines, such as IL-10 and TGF-*β*,^5^ which inhibit the maturation of DCs and their activity on CD8^+^ T cells, promoting tumor progression.^6^

We had previously reported^7^ that REG3A, the human homolog of mouse Reg3g, accelerated PDAC cell proliferation and tumor formation by activating an EGFR-mediated REG3A-JAK2/STAT3-positive feedback loop. We also found that, in mice, enhanced Reg3g expression exacerbated pancreatic cancer development in chronic pancreatitis through multiple mechanisms including suppression of T-lymphocyte proliferation and DCs function.^8^ The RegIII family comprises three secretion molecules: Reg3*α*, Reg3*β*, and Reg3g. Their expressions have been reported to be elevated in acute and chronic pancreatitis, inflammatory bowel disease, and liver injury.^9^ Recently, increasing attention has been paid to their overexpression in human cancer cells as well. Among the family members, Reg3*β* is present in PDAC and colorectal carcinoma.^10, 11^ Reg3*β* exhibited antiapoptotic effects and induced tumor-related macrophage polarization via activation of STAT3 signaling. This facilitated the production of tolerant CD4^+^ T cells, resulting in systemic tolerance and tumor immune escape.^12, 13^ We had reported^14^ that Reg3g promoted *β*-cell regeneration and postponed the emergence of type 1 diabetes by inducing Treg differentiation and inhibiting DCs maturation in type 1 diabetes. Reg family molecules are highly conserved in regulated innate immune cells involved in PDAC.^7^ Accordingly, we hypothesized that Reg3g enhances pancreatic tumorigenesis by associating with regulating tumor-associated innate immune cells to promote tumor immune escape. The results of this study generally support the hypothesis, revealing a novel mechanism for pancreatic tumor promotion by Reg3g.

## Results

### Reg3g enhanced pancreatic tumor growth and altered the composition of immune cells in the TME

**Figure 1:**
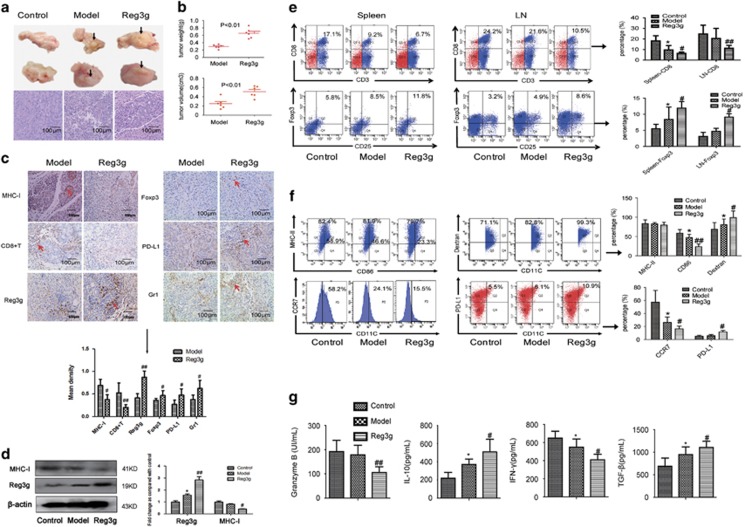
Reg3g promoted tumor growth in orthotopic pancreatic tumor mouse by unbalancing T-cell ratios and dendritic cell (DC) maturation. Panc02 cells were cultured with or without Reg3g lentiviral particles and implanted (2 × 10^5^/50 *μ*l) into the pancreas of C57BL/6 mice. Mice were killed and tumors were collected 21 days after injection. (**a**) Representative pictures of pancreatic tumors and pancreatic sections stained with hematoxylin–eosin in the control, model, and Reg3g groups. (**b**) Average tumor volumes and weights in each group. (**c**) Expressions of MHC-I, CD8, Reg3g, PD-L1, Gr-1, and Foxp3 in tumors were assessed by immunohistochemistry. They were quantificated by mean density, which was equal to integrated optical density of brown areas divided by whole areas. (**d**) The expression of MHC-I and Reg3g in tumors were analyzed by western blot. (**e**) The percentage of CD8^+^ T cells and regulatory T cells (Tregs) was detected by fluorescence-activated cell sorting (FACS) in the spleen and lymph node of control, model, and Reg3g mice. (**f**) The expression levels of CD86, MHC-II, CCR7, and PD-L1 on DCs surfaces, and the phagocytic capacity of DCs were measured by FACS. (**g**) Levels of granzyme B, IL-10, interferon-*γ* (IFN-*γ*), and tumor growth factor-*β* (TGF-*β*) in the serum were detected by enzyme-linked immunosorbent assay (ELISA). Data were presented as means±S.D. from at least three independent experiments. *P<0.05 compared with control; ^#^*P*<0.05, ^##^*P*<0.01 compared with the model group

To validate whether Reg3g, the mouse homolog of human REG3A, modulates pancreatic tumor growth via alteration of the TME composition *in vivo*, we established Reg3g-conditioned Panc02 cells, which exhibited upregulated Reg3g (Supplementary Figure S1a), and then implanted them into mice. Panc02 cells transfected by pEZ-Lv201-NEG^7^ showed no difference in Reg3g expression of Panc02 cells compared with control (Supplementary Figure S1a). However, Reg3g overexpression sped up the formation of pancreatic tumors as confirmed by histologic analysis (Figures 1a and b). Immunohistochemical and western blot analysis indicated that Reg3g overexpression recruited MDSCs marked with Gr-1 and Tregs marked with Foxp3, and upregulated the expression of PD-L1 (B7-H1) and MHC-I, but lowered the infiltration of CD8^+^ T cells in tumors. These results are consistent with the clinical data obtained from the GEO database that REG3A expression levels are elevated in human PDAC tissues (Figures 1c and d and Supplementary Figures S2a–c).

Flow cytometry analysis (FACS) revealed that Reg3g overexpression also decreased the population of CD3^+^CD8^+^ T cells, and augmented the frequency of Tregs in the spleen and lymph node (Figure 1e) of the orthotopic pancreatic tumor mice. These findings were consistent with results obtained from the GEO database that REG3A is negatively correlated with CD8A in lymph nodes of PDAC patients (Supplementary Figure S2f). In addition, Reg3g recruited MDSCs (CD11b^+^Gr-1^+^) and enhanced the expression of CTLA-4 and PD-1 in T cells, but hampered the expression of TCR (Supplementary Figure S3). We also found that Reg3g overexpression lowered CD86 expression, enhanced endo-/phagocytic function determined by the extent of FITC-dextran upregulation, inhibited migration assessed by CCR7 downregulation, and increased expression of PD-L1 (Figure 1f) in DCs from bone marrow of the orthotopic pancreatic tumor mice.

### Reg3g elicited Th2-mediated immunosuppression

Inflammation plays a critical role in tumorigenesis.^15^ The hallmarks of cancer-related inflammation include the presence of inflammatory cells and inflammatory mediators such as cytokines. We found that Reg3g overexpression decreased serum levels of the cytokines granzyme B and IFN-*γ*, but elevated TGF-*β* and IL-10 (Figure 1g). Consistent with its effects on cytokine secretion, Reg3g overexpression also reduced tumor expressions of mRNAs for Th1 cytokines IFN-*γ* and IL-12, and increased mRNAs for Th2 cytokines such as TGF-*β* and IL-10 (Figure 1g and Supplementary Figure S1b). Importantly, these results were consistent with previously observed effects of REG3A on IFN-*γ*, IL-12, and TGF-*β* secretion of human PDAC tissues (Supplementary Figure S2d). The diminished production of Th1 cytokines such as IFN-*γ* and IL-12 suggests that Reg3g inhibited both tumoricidal activity of CD8^+^ T cells and DC-induced antitumor immunity.

### Downregulation of Reg3g impaired pancreatic cancer tumor growth

**Figure 2:**
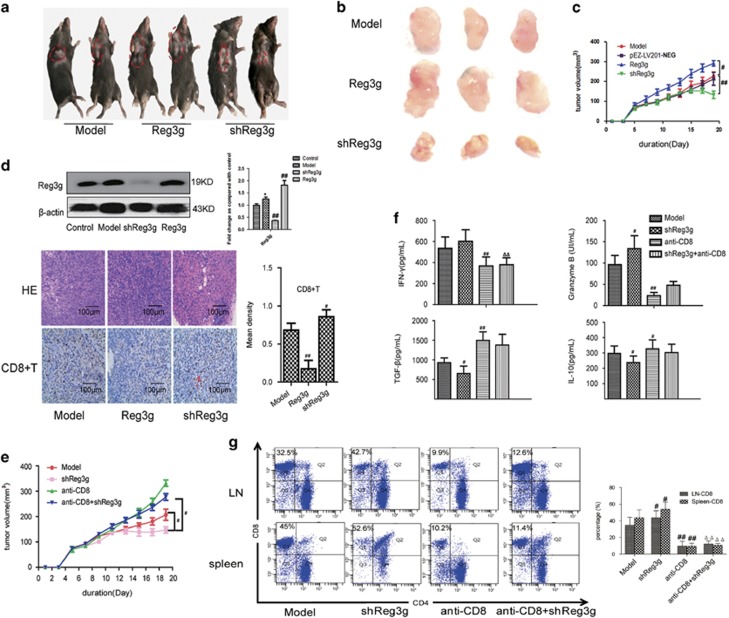
Reg3g increased tumor volume and decreased the percentage of CD8^+^ T cells. Various kinds of lentiviral particles were injected intraperitoneally into TBM as described in Materials and methods section. (**a**) Representative photographs of mice treated with PBS, Reg3g lentivirus, or shReg3g lentivirus. (**b** and **c**) Representative images of tumors and the average tumor volumes of mice in each group. (**d**) The expression of Reg3g in tumors was analyzed by western blot. In addition, representative pictures of tumors were analyzed by hematoxylin and eosin (H&E) staining, and the expression of CD8 in tumors was evaluated by immunohistochemistry. (**e**) Anti-CD8 antibody or shReg3g lentiviral particles was given intraperitoneally to TBM. Mice were killed and tumors were collected 19 days after injection. Average tumor volumes of mice in model, shReg3g, anti-CD8, and anti-CD8+shReg3g groups. (**f**) Levels of granzyme B, interleukin-10 (IL-10), interferon-*γ* (IFN-*γ*), and tumor growth factor-*β* (TGF-*β*) in the serum were detected by enzyme-linked immunosorbent assay (ELISA). (**g**) Representative fluorescence-activated cell sorting (FACS) staining for CD4 and CD8 on gated CD3^+^ T cells and the proportion of CD8^+^ T cells out of total CD3^+^ T cells in the spleen and lymph nodes isolated from TBM. Data were expressed as means±S.D. from at least three independent experiments. ^#^*P*<0.05, ^##^*P*<0.01 compared with the model group; ^Δ^*P*<0.05, ^ΔΔ^*P*<0.01 compared with shReg3g

To further characterize the role of Reg3g in tumor growth, we treated tumor-bearing mice (TBM) with shReg3g or pReg3g lentiviral particles to induce Reg3g downregulation or overexpression, confirmed by western blot (Figure 2d). The results showed that Reg3g downregulation inhibited tumor growth, whereas Reg3g overexpression increased tumor growth (Figures 2a–c). In the controls, pEZ-Lv201-NEG had no effect on tumor growth compared with the model group (Figure 2c). Histologic studies revealed that there were areas of vacuoles and necrosis in tumor tissue of shReg3g-treated mice (Figure 2d). Treatment with Reg3g lentivirus diminished the amount of CD8^+^ T cells in tumors of TBM, whereas shReg3g treatment increased it (Figure 2d).

### Reg3g restrained tumoricidal activity of CD8^+^ T cells and DCs maturation

To further investigate the role of CD8^+^ T cells in Reg3g-mediated tumor progression, we treated TBM with anti-CD8 mAb or shReg3g lentiviral particles. We found that deficiency of CD8^+^ T cells in TBM resulted in a significant increase in tumor volumes. Suppression of Reg3g failed to inhibit tumor growth in the absence of CD8^+^ T cells (Figure 2e). The absence of CD8^+^ T cells in the spleen and lymph node (Figure 2g) was accompanied by lower serum levels of granzyme B and IFN-*γ* and elevated levels of IL-10 and TGF-*β* (Figure 2f). The data indicated that downregulation of Reg3g inhibited tumor growth, and that CD8^+^ T-cell deletion abolished the effect.

**Figure 3:**
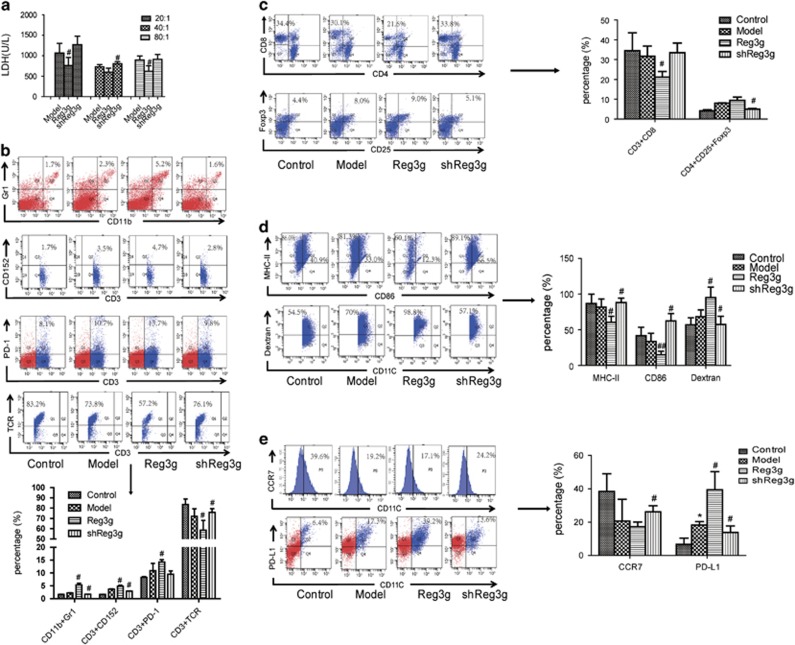
Reg3g downregulation enhanced dendritic cell (DCs) maturation and antitumor effect of T cells. (**a**) CD8^+^ T cells cocultured with Panc02 cells in the ratio of 20 : 1, 40 : 1, and 80 : 1 for 24 h. The cytotoxic ability of CD8^+^ T cells were then detected by the release of lactic dehydrogenase (LDH). (**b**) The proportion of CD11bGr1, CD3PD-1 in the spleen and CD3CD152, CD3TCR on CD3^+^ T cells gating in the spleen were measured by fluorescence-activated cell sorting (FACS). (**c**) The percentage of CD8^+^ on CD3^+^ T cells gating in the spleen and CD4^+^CD25^+^Foxp3^+^Treg on CD4^+^ T cells gating in the spleen were detected by FACS. (**d** and **e**) The expression levels of CD86, MHC-II, and CCR7 on CD11C^+^ cells and CD11CPD-L1 on DCs, and phagocytic function (the percentage of FITC-dextran in CD11C^+^ cells) of DCs were all assessed by FACS. Data were presented as means±S.D. from at least three independent experiments.*P<0.05 compared with control; ^#^*P*<0.05, ^##^*P*<0.01 compared with the model group

We also found that Reg3g overexpression robustly suppressed cytotoxic T-lymphocyte (CTL) activity, especially at a CD8^+^ T-cell: Panc02 ratio of 20 : 1 (Figure 3a). In addition, the number of tumor-infiltrating MDSCs and Tregs and immunosuppressive markers of CTLA-4 and PD-1 on T cells were suppressed in shReg3g mice, but the frequency of CD8^+^ T cells and the expression of TCR in T cells were increased (Figures 3b and c). Thus, Reg3g overexpression and suppression produced opposite effects on PD-L1 expression and DC maturation, migration, and endo/phagocytic function (Figures 3d and e).

### Reg3g overexpression suppressed the cross-priming of DCs and CD8^+^ T-cell responses by activating the JAK2/STAT3 signaling pathway

**Figure 4:**
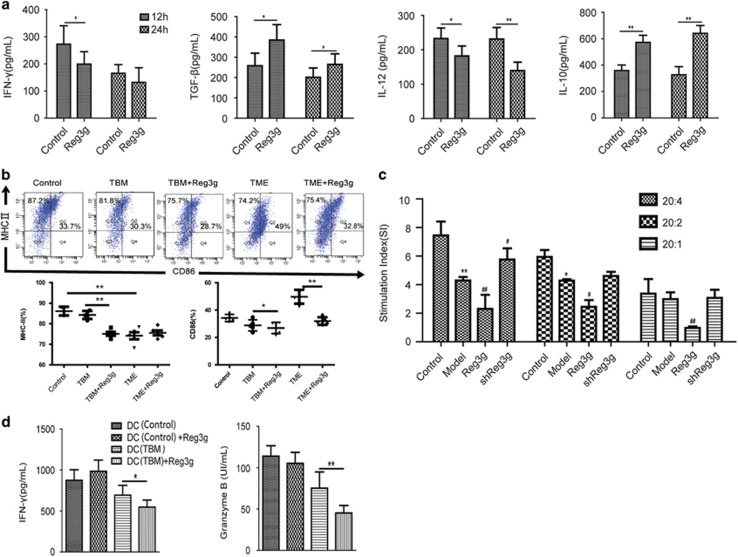
Reg3g inhibited dendritic cell (DC) maturation and cross-priming with CD8^+^ T cells in TME. (**a**) DCs from TBM were coincubated with 100 ng/ml Reg3g for 12 or 24 h. The secretions of interferon-*γ* (IFN-*γ*), tumor growth factor-*β* (TGF-*β*), interleukin-12 (IL-12), and IL-10 in DC supernatants were quantified by enzyme-linked immunosorbent assay (ELISA). (**b**) The expression of CD86 and MHC-II on CD11C^+^ DCs from control mice, TBM, and TBM treated with Reg3g lentiviral particles (TBM+Reg3g). In addition, culture medium from Panc02 cells (TME), and 100 ng/ml Reg3g+TME, were added to DCs from control mice. The expression of CD86 and MHC-II on DCs was determined by fluorescence-activated cell sorting (FACS). (**c**) T cells were cocultured with DCs at the ratio of 20 : 1, 20 : 2, and 20 : 4 for 24 h. The proliferation of T cells was then determined by 3-(4,5-dimethylthiazol-2-yl)-2,5-diphenyltetrazolium bromide (MTT) assay. (**d**) CD8^+^ T cells were cocultured with DCs from different groups for 24 h, and levels of granzyme B and IFN-*γ* in the supernatant were determined by ELISA. Data were shown as means±S.D. from at least three independent experiments. *P<0.05, **P<0.01 compared with control; ^#^*P*<0.05, ^##^*P*<0.01 compared with the model group

To characterize effects of Reg3g on the maturation and function of DCs, we collected DCs from TBM and incubated them with 100 ng/ml Reg3g for 12 and 24 h. The effective dose of Reg3g had been established in a previous study (data not shown). Reg3g increased the production of the anti-inflammatory cytokines TGF-*β* and IL-10, but inhibited the production of the inflammatory cytokines IFN-*γ* and IL-12 (Figure 4a). To further investigate how Reg3g regulated tumor inflammatory responses through DCs, we isolated DCs from control, TBM, and Reg3g-overexpressed TBM. The expressions of MHC-II and CD86 on DCs were lower in the Reg3g-overexpressed TBM compared with the TBM (Figure 4b). When we cultured DCs with conditioned medium from Panc02 cells, mimicking TME, we found that Reg3g suppressed CD86 on DCs (Figure 4b) *in vitro* as well.

To determine effects of Reg3g overexpression on the presentation of antigens by DCs to T cells, the stimulatory capacity of DCs was assessed by the mixed leukocyte reaction (MLR). DCs from Reg3g-overexpressed TBM effectively restricted responder T-cell proliferation, even at very low DC–T-cell ratios (Figure 4c). We next collected DCs from control mice or TBM in the presence or absence of Reg3g and cocultured them with CD8^+^ T cells from control mice. As predicted, we found that Reg3g suppressed the secretion of IFN-*γ* and granzyme B from CD8^+^ T cells, consistent with low numbers and immunostimulatory activities of CD8^+^ T cells, in some cases reaching levels below those of the controls (Figure 4d).

**Figure 5:**
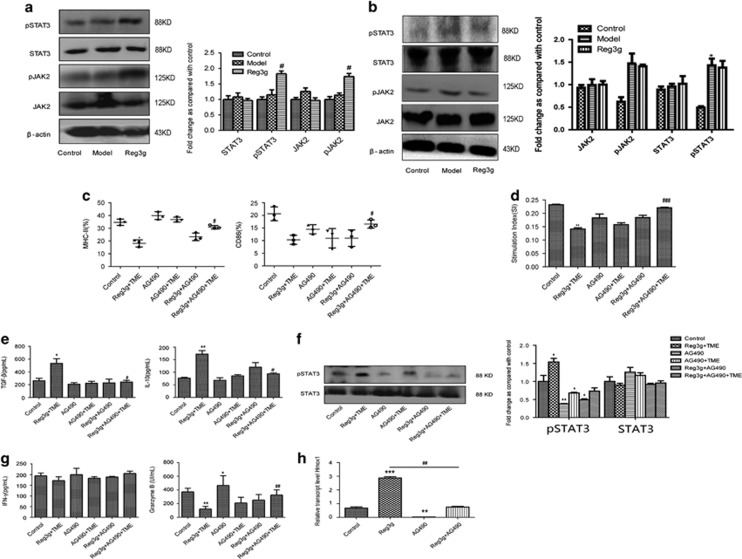
Reg3g inhibited dendritic cells (DCs) by activating /janus kinase 2/signal transducer and activator of transcription 3 (JAK2/STAT3) signaling pathway. (**a**) The expressions of phosphorylated (p)STAT3, STAT3, JAK2, and pJAK2 proteins in DCs were analyzed by western blot. (**b**) The expression of pSTAT3, STAT3, pJAK2, and JAK2 were analyzed by western blot in CD8^+^ T cells separated using a negative CD8 Isolation Kit (Aubum, CA, USA) from the spleen of control, model, and Reg3g mice. (**c** and **e**) DCs were treated with Reg3g+TME, AG490, AG490+TME, Reg3g+AG490, and Reg3g+AG490+TME, respectively. Fluorescence-activated cell sorting (FACS) analysis for the quantitative percentage of CD86 and MHC-II, and the production of interlukin-10 (IL-10) and tumor growth factor-*β* (TGF-*β*) in the culture medium were detected by enzyme-linked immunosorbent assay (ELISA). (**d**) CD8^+^ T cells were cocultured with DCs at the ratio of 10:1 for 24 h. The proliferation of T cells was then evaluated by the 3-(4,5-dimethylthiazol-2-yl)-2,5-diphenyltetrazolium bromide (MTT) method. (**f** and **g**) The protein expressions of STAT3 and pSTAT3 were identified by western blot. The levels of interferon-*γ* (IFN-*γ*) and granzyme B in culture medium were estimated by ELISA. (**h**) The mRNA level of Hmox1 in DCs was analyzed by qRT-PCR. Data were shown as means±S.D. from at least three independent experiments. *P<0.05, **P<0.01, ***P<0.001 compared with the control group; ^#^*P*<0.05, ^##^*P*<0.01, ^###^*P*<0.001 compared with the model group (**a** and **b**); ^#^*P*<0.05, ^##^*P*<0.01 compared with Reg3g+TME group (**c**–**g**)

Interestingly, we observed that Reg3g overexpression strongly activated pSTAT3 and pJAK2 in DCs, resulting in inhibition of DC maturation (Figure 5a). However, Reg3g overexpression had no significant effect on the basal JAK2/STAT3 pathway in CD8^+^ T cells (Figure 5b). Consistent with results obtained from MLR described above, these results imply that Reg3g initially suppressed maturation of DCs and subsequently interrupted cross-priming of CD8^+^ T-cell responses, ultimately contributing to the development of an aberrant tumor immune microenvironment.

Furthermore, we found that suppression of JAK2 by AG490 in DCs alleviated the inhibitory effect of Reg3g on the maturation of DCs, and strongly attenuated the expression of downstream gene *Hmox1* and the secretion of IL-10 and TGF-*β* in TME (Figures 5c, e, f, and h). Further, in the presence of Reg3g, downregulation of JAK2 in DCs decreased the expression of STAT3 protein and promoted the proliferation and the secretion of IFN-*γ* and granzyme B of CD8^+^ T cells in the TME (Figures 5d and g). Overall, the results are consistent with the view that Reg3g suppresses T-cell proliferation and CTLs by activating the JAK2/STAT3 pathway in DCs, inhibiting their immunostimulatory activities in the TBM.

### Reg3g exerted an indirect functional effect on Panc02 cells by inhibiting DCs maturation *in vitro*

**Figure 6:**
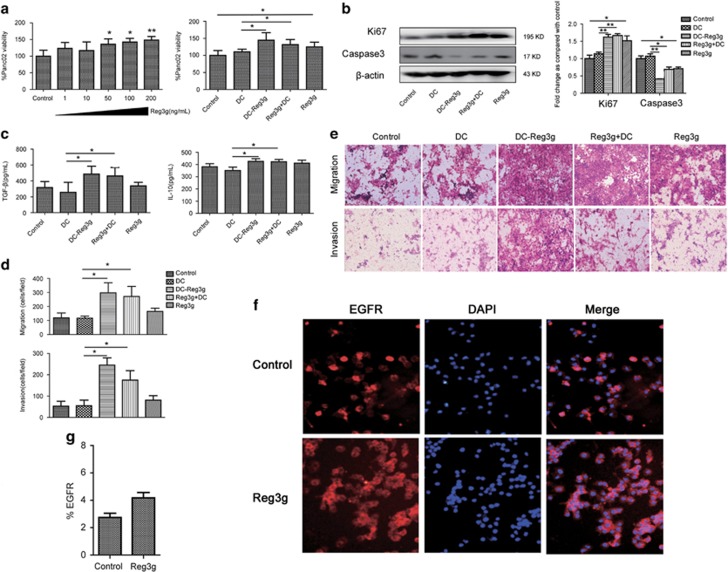
Reg3g exerted an indirect functional effect on Panc02 cells via dendritic cell (DC) maturation *in vitro*. (**a** and **b**) After treatment of Panc02 cells with Reg3g in a dose-dependent manner (1–200 ng/ml) for 24 h, Panc02 cells were incubated with DCs for 24 h. Cell viability was determined using the Cell Counting Kit-8 (CCK-8) assay. Expression of caspase-3 and Ki67 were determined by western blot. (**c**) Levels of interleukin-10 (IL-10) and tumor growth factor-*β* (TGF-*β*) in Panc02 cells supernatant were measured by enzyme-linked immunosorbent assay (ELISA). (**d** and **e**) Migration and invasion of Panc02 cells were assessed by crystal violet staining. Indexes of migration and invasion were calculated using a Computerized Image Analysis System (ImageJ, Bethesda, MD, USA). (**f**) Epidermal growth factor receptor (EGFR) (red) level in DCs were determined by immunofluorescent staining. Control: DCs from TBM; Reg3g: DCs from TBM in combination with 100 ng/ml Reg3g. (**g**) Quantitative assessment of EGFR expression in DCs. Data were represented as means±S.D. from at least three independent experiments. *P<0.05, **P<0.01 compared with the control group

On the basis of the *in vitro* results—that Reg3g inhibited DCs maturation in TME—we further investigated whether Reg3g influenced growth, cytokine secretion, migration, or invasion of Panc02 cells directly, or indirectly through its effects on DC maturation. Reg3g administration (0–200 ng/ml) dose-dependently increased Panc02 cell proliferation (Figure 6a). A concentration of 100 ng/ml was then selected for further investigations of effects of Reg3g on Panc02 cell growth.

Panc02 cells were incubated with conditioned media from four groups: (1) DC; (2) DC-Reg3g; (3) Reg3g+DC; and (4) Reg3g. Incubation with conditioned media from either DC-Reg3g or Reg3g+DC increased the viability of Panc02 cells (Figure 6a), enhanced their expressions of Ki67, decreased their expressions of caspase-3 (Figure 6b), and promoted the secretion of IL-10 and TGF-*β* and migration and invasion of Panc02 cells (Figures 6c–e). Reg3g also promoted the expression of EGFR in the cytoplasm and membranes of DCs (Figures 6f and g). In a previous report from this laboratory,^7^ EGFR had been identified as a mediator in the Reg3g/STAT3 pathway. We observed here that Reg3g promoted the expression of pSTAT3 in DCs, and that EGFR interference with an EGFR-specific inhibitor (Erlotinib HCL) inhibited the effect (Supplementary Figure S5a), confirming that EGFR mediated the activity of Reg3g.^7^

Notably, increasing concentrations of anti-Reg3g did not affect the viability of Panc02 cells (Supplementary Figure S4a). In fact, neutralization of Reg3g protein by anti-Reg3g for 24 h, at the ratio of 1:1000, appeared to increase Panc02 cell viability compared with the viability of untreated DCs, but the difference was not significant compared to DCs with Reg3g (Supplementary Figure S4b). Overall, the results confirmed that Reg3g indirectly promoted the proliferation of Panc02 cells by acting on DCs with immature phenotypes, increasing the secretion of IL-10 and TGF-*β*.

### Reg3g activates the EGFR/JAK2/STAT3 signaling pathway in tumors

**Figure 7:**
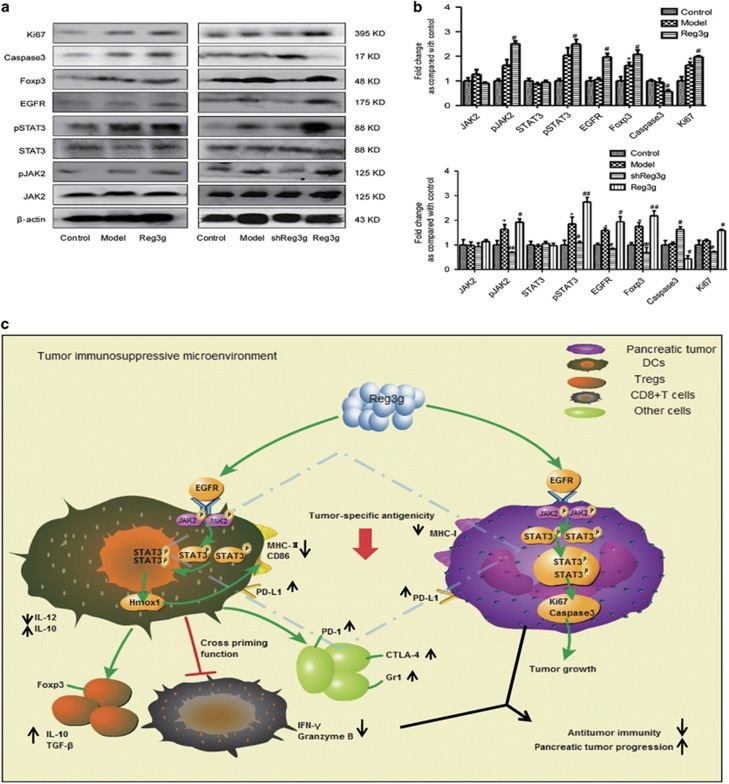
Reg3g activated epidermal growth factor receptor/janus kinase 2/signal transducer and activator of transcription 3 (EGFR/JAK2/STAT3) signal pathway in tumors. (**a**) Expressions of phosphorylated (p)STAT3, STAT3, pJAK2, JAK2, Foxp3, caspase-3, Ki67, and EGFR were analyzed by western blot in tumors of orthotopic (the left) or ectopic (the right) pancreatic tumor mice. (**b**) The column chart was a quantitative analysis of the western blot corresponding to (**a**). (**c**) Reg3g promoted pancreatic tumor progression and inhibited antitumor immunity by triggering the activation of STAT3 signaling. Data were represented as means±S.D. from at least three independent experiments. *P<0.05, compared with the control group; ^#^*P*<0.05, ^##^*P*<0.01 compared with the model group

According to our previous study,^7^ EGFR, functioning as a REG3A receptor, mediated the REG3A signal-promoting human pancreatic cancer cell growth and JAK2/STAT3 activation. Here, we detected EGFR/JAK2/STAT3 components in the implanted tumors and found that Reg3g overexpression upregulated the expression of EGFR, pJAK2, pSTAT3, Ki67, and Foxp3, but inhibited the level of caspase-3 in both orthotopic and ectopic xenograft tumors (Figures 7a and b). As expected, Reg3g knockdown produced opposite results. To confirm that EGFR-driven STAT3 signaling is one of the key mechanisms, we added an EGFR-specific inhibitor to Panc02 cell media. The inhibitor decreased the expression of pSTAT3. This response was not reversed by Reg3g (Supplementary Figure S5b). These data indicated that Reg3g activated the EGFR/JAK2/STAT3 signal pathway in tumor cells, confirming results of our previous study.^7^

## Discussion

The results of this study support the view that Reg3g plays a crucial role in accelerating PDAC progression^16^ by altering the TME. Specifically, Reg3g promotes proliferation, migration, invasion, and secretion of cytokines by Panc02 cells by inhibiting DCs maturation. Our study is thus consistent with the report that inhibiting the maturation of DCs and disrupting the cross-priming of DCs and T cells contribute to tumor growth and tumor-promoting microenvironment formation.^17^

Reg3g is a soluble small protein, rarely secreted in the normal pancreas, but markedly overexpressed during acute or chronic pancreatitis. Its overexpression is marked by elevated levels of pancreatitis-associated protein.^18^ Our group has previously characterized the promotion by Reg3g of *β*-cell regeneration and immunosuppression in type 1 diabetes.^14^ We also reported that Reg3g accelerated malignant transformation in chronic pancreatitis and promoted pancreatic carcinogenesis partly via immunosuppressive action.^8^

We analyzed the GEO data sets, which revealed that increases of REG3A in PDAC tissues were associated with fewer CD8A and T-cell cytokine imbalances. This is in agreement with our previous study showing that REG3A accelerated tumor growth via STAT3 signaling, and with our present findings indicating that Reg3g promoted tumor growth by suppressing immune responses.

Therefore, we established orthotopic or ectopic mouse models of pancreatic cancer, and found that Reg3g overexpression promoted tumor growth by accelerating Ki67 and inhibiting the cell apoptosis-related protein caspase-3. These changes were followed by increased production of Tregs, immature DCs, and MDSCs along with higher levels of TGF-*β* and IL-10 and reduced CD8^+^ T cells infiltration in tumors. Further, 'Silencing' Reg3g increased the percentage of effector CD8^+^ T cells around the tumors and improved their antitumor effects. The absence of CD8^+^ T cells in TBM abolished the effect. In addition, treatment with Reg3g promoted the upregulation of PD-1 in T cells, and of PD-L1 and EGFR in tumors, and decreased the expression of TCR in T cells and MHC-I in tumors. These changes endowed tumors with less antigenicity,^19, 20^ resulting in decreased numbers of DCs and CD8^+^ T cells involved in immune responses.

The TME of pancreatic cancer supports malignant progression of tumors. It is characterized by recruitment of immune cells including T cells, macrophages, granulocytes, and DCs.^21^ Hirooka *et al.*^22^ clarified the role of DCs in the initiation and modulation of immune system responses. Patients with higher numbers of infiltrating mature DCs in resectable pancreatic cancer have more favorable outcomes.^23, 24^ DCs are potent antigen-presenting cells, and act as intermediaries between the tumor and the resultant T-cell response.^25, 26^ They are also involved in cross-priming CD8^+^ T cells,^27, 28^ which adds to the immune response by enhancing the expressions of MHC-II.^29^ Here, we report that Reg3g suppressed DC maturation markers MHC-II and the costimulatory molecule CD86, both in TBM and TME induced by administration of Panc02 cell-conditioned medium. This was consistent with previous reports^30, 31^ that tumor cells were able to avoid immunosurveillance by producing an immunosuppressive environment that inhibits maturation and function of DCs.

We also found that Reg3g overexpression suppressed the presentation of antigens by DCs to CD8^+^T cells, and Reg3g-treated DCs effectively impaired responder T-cell proliferation, even at very low ratios of DCs: T cells. This corresponded with an increase in the accumulation of effective tumor association antigens and a decrease in tumor-specific T-cell activation. Lower levels of IFN-*γ* and Granzyme B secretion were associated with a decline in cytotoxic activity of CD8^+^T cells. In addition, we also found that Reg3g elevated interactions between inhibitor ligand PD-L1 in DCs and PD-1 in T cells, resulting in the suppression of tumor-specific T-cell activation. Thus, Reg3g seems to suppress cytotoxic activities of CD8^+^T cells by regulating the maturation and function of DCs, thereby blunting immune responses and promoting tumor growth.

Cytokines derived from immune cells or cancer cells also play key roles in tumor immune modulation. For example, the level of IL-10 in tumors is correlated with induction and activation of Tregs and MDSC.^32, 33^ Decreased IL-12 expression and DC maturation inhibits CD8^+^ T-cell-dependent antitumor immune responses.^34^ Tissue and serum levels of IL-10 are elevated in PDAC patients.^30, 35^ In this study, we found that Reg3g treatment of TBM increased the percentages of Th2 cytokines such as IL-10 and TGF-*β*. Conversely, Reg3g treatment of orthotopic pancreatic tumor mice decreased levels of Th1 cytokines such as IFN-*γ*, IL-12, and TNF-*α*. The immunosuppressive microenvironment may thus permit the pancreatic cancer cells to escape detection and destruction.

It is a matter of debate as to whether Reg3g promotes the proliferation, cytokine secretion, migration, and invasion of Panc02 cells directly, or indirectly by influencing interactions between DCs and tumor cells. In this study, we confirmed that Reg3g promoted Panc02 cell proliferation and inhibited its apoptosis directly (Figures 6a and b). However, we also provide evidence supporting the second possibility. Conditioned media from Reg3g-treated DCs of TBM inhibited apoptosis and promoted Panc02 cell growth, consistent with *in vivo* results showing tumor enhancement induced by Reg3g overexpression. Either conditioned media from DCs of Reg3g-overexpressed TBM or conditioned media from Reg3g-treated DCs stimulated the secretion of IL-10 and TGF-*β*, migration, and invasion of Panc02 cells. These results confirmed that Reg3g hampered the maturation of DCs, consequently producing immunosuppressive conditions in the TME by increasing the secretion of IL-10 and TGF-*β* and inhibiting CTL. To determine whether indirect effects of Reg3g involves altering functions of cells other than DCs will require additional studies.

Of interest, we found that Reg3g promoted EGFR expression in DCs. We previously reported that EGFR mediated the REG3A signal for PDAC cell growth and JAK2/STAT3 activation, thus functioning as a REG3A receptor.^7^ We suggested that Reg3g inhibited the maturation and function of DCs by activating EGFR and its downstream STAT3 signaling in DCs. Our further study revealed that Reg3g upregulated *Hmox1*, a downstream gene of Reg3g/STAT3 signaling. As Hmox1 is not only an effector of the JAK2/STAT3 pathway but also is an inhibitor of DCs maturation,^36, 37^ our results indicated that Reg3g inhibited the maturation of DCs at least in part by acting through the JAK2/STAT3 pathway. It was reported that STAT3 activation in myeloid cells increased anti-inflammatory actions of IL-10, whereas deletion of STAT3 resulted in severe enterocolitis.^38^ The inhibitory effects of IL-6 on DCs differentiation could be partially prevented by inhibition of STAT3 by the JAK2 inhibitor AG490.^39^ In STAT3-deficient mice,^40^ STAT3-dependent regenerating genes Reg3*β* and Reg3g are also deleted, while colonic expression of SOCS3, a direct downstream gene of STAT3, is significantly increased. Consistent with these observations, our data here showed that activation of STAT3 signaling by Reg3g in DCs enhanced the secretion of IL-10 and TGF-*β*, resulting in T-cell imbalance and inhibition of antitumor immune responses by CD8^+^ T cells.

In summary, our findings reveal an important and novel tumor-promoting role of Reg3g in PDAC. We postulate that the overexpression of Reg3g increases EGFR, but suppresses tumor antigenicity by upregulating the levels of Ki67 and inhibiting the expression of caspase-3 and MHC-I in tumors. Reg3g also suppresses the maturation of DCs via activation of the Hmox1 involved in JAK2/STAT3 signaling pathway, which weakens cytotoxic activities of CD8^+^ T cells by promoting PD-1 and PD-L1 expressions and interactions between cells. Ultimately, Reg3g fosters a tumor environment consisting of Th2 cytokines IL-10 and TGF-*β* and of Tregs and MDSCs, accelerating tumor growth (Figure 7c).

## Materials and methods

### Cell line and mice

The murine pancreatic adenocarcinoma cell line Panc02, syngenic to C57BL/6 mice, was purchased from Shanghai Aolu Biological Technology Co. Ltd (Shanghai, China). Panc02 cells were maintained at 37 °C under a 5% CO_2_ environment in DMEM (Hyclone, Logan, UT, USA) enriched with 10% FBS and penicillin–streptomycin (100 U/ml) (Invitrogen, Carlsbad, CA, USA).

Sixty male C57BL/6 mice at the age of 6 weeks were purchased from Beijing HFK Bio-Technology Co. Ltd (Beijing, China). The mice were purchased, shipped, housed, cared for, and killed according to guidelines provided by the Institutional Animal Care and Use Committee of Tongji Medical College.

### Orthotopic or ectopic pancreatic tumor mouse model of pancreatic cancer

*Orthotopic mouse model*: We transfected Panc02 cells by pEZ-Lv201-Reg3g^7^ (pReg3g) to construct Reg3g-conditioned Panc02 cells. PEZ-Lv201-Reg3g was composed of pEZ-Lv201 mammalian gene expression vector system and a target gene *Reg3g* to build an overexpression system of Reg3g. Panc02 (2 × 10^5^, Model group) or Reg3g-overexpressed Panc02 cells (2 × 10^5^, Reg3g group) were transplanted into the tail of pancreas followed the approach.^12^ Mice with tumor-free mice were regarded as control. All mice were killed after 3 weeks of treatment.

*Reg3g-conditioned Panc02 cells*: In brief, we added 10^7 ^TU/ml of pReg3g lentivirus to fresh RPMI 1640 culture medium in the presence of 8 *μ*g/ml polybrene (Sigma, St. Louis, MO, USA) when Panc02 cells were grown up to 50% in T25 flask (~2.5 × 10^6^ cells). About 48 h post infection, the medium was replaced with medium containing 2 *μ*g/ml puromycin (Sigma) for 24 h to make Reg3g-conditioned Panc02 cells. And, the MOI of Panc02 cells represent the ratio of input infectious units of lentivirus to the number of cells available is 4.

*Ectopic mouse model*: Panc02 cells (2 × 10^6^cells per mouse) were subcutaneously implanted into the flank of C57BL/6 mice, designated TBM. After 5 days, the growth of the tumor was detected, and mice were randomly divided into pReg3g and psi-LVRU6MP-shReg3g (shReg3g) group.^7^ Meanwhile, the Reg3g group was administered 0.1 ml PBS with 1 × 10^7^ TU/ml of pReg3g lentiviral particles intraperitoneally (GeneCopoeia, Rockville, MD, USA), and the other group was given corresponding lentiviral particles intraperitoneally. Psi-LVRU6MP-shReg3g is a lentiviral expression vector that expresses shRNA to downregulate mouse Reg3g. All of the mice were killed after 2 weeks of treatment.

*Anti-CD8 mAbs blocking*: Panc02 (2 × 10^6^) cells were subcutaneously implanted into the flank. At the same time, 200 *μ*g of anti-CD8 antibody (clone TIB210) was injected intraperitoneally. Then, tumors were allowed to grow for 5 days and treated by corresponding shReg3g lentiviral particles intraperitoneally.

Tumor size was measured every other day and the tumor volume was calculated as follows: tumor volume (mm^3^)=(length × width^2^) × 0.5.

### Cytokine measurement

The serum concentrations or cell culture supernatant of cytokines including mouse IL-12, IL-10, TGF-*β*, granzyme B, and IFN-*γ* were determined by ELISA Kits (eBioscience, San Diego, CA, USA).

### Flow cytometry analysis

T-cell suspensions were isolated from the spleen and lymph nodes, and were stained with antibodies against the following cell surface antigens: APC-conjugated anti-mouse CD3, FITC-conjugated anti-mouse CD4, PE-conjugated anti-mouse CD8, PE-conjugated anti-mouse Gr-1, APC-conjugated anti-mouse CD11b, PE-conjugated anti-mouse TCR (eBioscience), PE-conjugated anti-mouse PD-1, and PE-conjugated anti-mouse CD152 (CTLA-4; BD Bioscience, San Jose, CA, USA). Tregs were detected from the spleen and lymph nodes using a Mouse Regulatory T-Cell Staining Kit (eBioscience) according to the manufacturer's instructions. DCs were isolated from the bone marrow and cells were incubated with FITC-conjugated anti-CD86, APC-conjugated anti-CD11C, PE-conjugated anti-MHC-II, FITC-dextran, PE-conjugated anti-CCR7, and anti-PD-L1 (eBioscience). The cells were analyzed by FACS, and the acquired data was performed with FlowJo Software, version 9.1 (Tree Star, San Carlos, CA, USA).

### Generation of DCs from bone marrow cells

DCs were isolated from murine bone marrow precursors as reported previously.^41^ In brief, 2 × 10^7^ cells were cultured in 12-well plates, which were supplemented with RPMI 1640 medium (HyClone, Beijing, China) added 10% FBS and GM-CSF (20 ng ml^−1^; SAB, Pearland, TX, USA) and IL-4 (10 ng ml^−1^; SAB). The fresh medium containing GM-CSF and IL-4 was added every other day. After 7 days, the cells were harvested for MLR and FACS.

### Measurement of tumoricidal activity of CD8^+^ T cells and MLR

CD8^+^ T cells were isolated from spleens of control mice using a negative CD8^+^ T-Cell Isolation Kit (MACS, Miltenyi Biotec Inc., Aubum, CA, USA). DCs obtained from control mice or TBM were incubated with or without Reg3g protein (SAB), and then were cocultured with CD8^+^ T cells at the ratio of 1 : 10 for 48 h (control, control+Reg3g, TBM, and TBM+Reg3g groups). Levels of granzyme B and IFN-*γ* in cell culture supernatants were detected by ELISA. For cytotoxic T-lymphocyte (CTL) assays, CD8^+^ T cells were cocultured with Panc02 cells for 24 h (the ratios were 20 : 1, 40 : 1, and 80 : 1), and the secretion of LDH in the cell culture supernatant was detected by a commercial kit (Nanjing Jiancheng Bioengineering Institute, Nanjing, China).

*MLR*: DCs from different groups of mice were treated with 0.5 mg/ml mitomycin C (Sigma, St. Louis, MO, USA) to prevent proliferation, and cocultured with T cells from the spleen of control mice in the ratio of 1 : 20, 2 : 20, and 4 : 20 for 24 h. Immunostimulatory activities of DCs were detected by MTT assay (Amersco, Solon, OH, USA).

### Western blot analysis, immunohistochemistry, and qRT-PCR

Cells or tissues were lysed, separated, and transferred onto polyvinylidene difluoride membranes. Membranes were incubated overnight at 4 °C with a 1 : 1000 dilution of anti-Reg3g (SAB), EGFR (SAB), Ki67 (Santa Cruz, Santa Cruz, CA, USA), pSTAT3 (Ser727), caspase-3, Foxp3, MHC-I (SAB), anti-STAT3, JAK2, pJAK2 (Tyr1007/1008) (CST, Boston, MA, USA), HRP-conjugated secondary antibodies were applied for 1–2 h at room temperature. Proteins were detected with an ECL Chemiluminescence Detection Kit (Advansta, Menlo Park, CA, USA).

Pancreatic tumors were fixed and embedded in paraffin. Sections were blocked and incubated with mouse PD-L1, Gr-1, MHC-I, CD8, Foxp3, and Reg3g antibody. Slides were washed and incubated with horseradish peroxidase-labeled anti-rabbit secondary antibody. Later, slides were stained with H&E by standard procedures and were visualized under microscope, and photographed.

Total RNA was isolated from tissues and cells with the MagZol reagent (Magen, Shanghai, China). Then, first-strand cDNA was generated from the extracted total RNA by All-in-one First-Strand cDNA Synthesis Kit (FulenGen, Guangzhou, China). Quantitative real-time PCR (qRT-PCR) was performed with 2.5 *μ*l of cDNA production in a 20 *μ*l reaction mixture using All-in-one qPCR Mix (FulenGen) with 3′ and 5′ primer at concentrations of 10 *μ*mol^−^^1^. QRT-PCR was carried out in an ABI 7900 Real-Time PCR System (LineGenek, Bioer Technology Co., Ltd, Hangzhou, China), with all target gene primers displayed in the Supplementary Material (Supplementary Table S1). The mRNA levels of target genes were normalized using detection of *β*-actin.

### Cell migration and invasion assay

The *in vitro* migration and invasion assay was performed using a 24-well transwell chamber (8 *μ*m pore size; Corning Costar, Cambridge, MA, USA, cat. no., 23510037) with a polycarbonate membrane. The inserts were precoated with 30 *μ*g of Matrigel (BD Bioscience) in the invasion assay. Panc02 cells (5 × 10^4^ cells/ml) were starved in serum-free media overnight, and then introduced into the upper chamber. The basolateral chamber was filled with the RPMI 1640 medium with 10% FBS. Cells were allowed to migrate through polycarbonate transwell inserts (Corning, Shanghai, China) for 12 h (migration) or 24 h (invasion) at 37 °C. Non-migrating or non-invading cells were removed from the top chamber by using a cotton swab. The cells remaining in the bottom chamber were fixed with 4% paraformaldehyde for 15 min and stained with 1% crystal violet in 2% ethanol for 20 min. Migrated cells were counted under the microscope (IX51, Olympus, Tokyo, Japan) in three random fields.^7^

### Cell growth and TME formation

Panc02 cells were incubated with:

1. media alone (control); conditioned media from DCs of TBM (DC); conditioned media from DCs of Reg3g-treated TBM (DC-Reg3g); conditioned media from DCs of TBM in combination with 100 ng/ml Reg3g (Reg3g+DC); and 100 ng/ml Reg3g alone for 24 h to determine cell viability, secretion of IL-10, and TGF-*β*, migration and invasion assessed by crystal violet staining.^7^ All experiments were performed in duplicate for each sample.
2. media alone (control); incubated with 50 *μ*M Erlotinib HCL (EGFR-specific inhibitor; Selleck, Houston, TX, USA) for 24 h (Erlotinib); incubated with 100 ng/ml Reg3g for 24 h (Reg3g); incubated with 50 *μ*M Erlotinib HCl and 100 ng/ml Reg3g for 24 h (Erlotinib+Reg3g).

DCs from TBM were exposed to:

1. PBS; 100 ng/ml Reg3g, respectively.
2. media alone (control); incubated with 50 *μ*M Erlotinib HCL (EGFR-specific inhibitor; Selleck) for 24 h (Erlotinib); incubated with 100 ng/ml Reg3g for 24 h (Reg3g); incubated with 50 *μ*M Erlotinib HCL and 100 ng/ml Reg3g for 24 h (Erlotinib+Reg3g).

DCs from control mice were exposed to: TME, 10% Panc02-conditioned medium; and Reg3g+TME, respectively. DCs were treated with 10 *μ*M AG490 (Sigma) for 6 days to suppress the expression of STAT3.

### Reg3g neutralization by antibody

The conditioned media from DCs of TBM were treated with 100 ng/ml Reg3g for 24 h. To get rid of the interference of Reg3g protein, we adopted the anti-Reg3g to neutralize the Reg3g protein. In brief, we added anti-Reg3g (SAB) to the conditioned media treated with Reg3g protein in the ratio of 1 : 10 000, 1 : 5000, 1:2500, 1 : 1000, 1 : 500, and 1 : 250 for 8 h, 12 h, and 24 h, respectively. Then, 10% conditioned media incubated with Panc02 cells for 24 h, respectively, to determine cell viability assessed by crystal violet staining.

### Bioinformatics analysis

We retrieved the data set from the NCBI GEO databank under accession number GSE28735, GSE62452 and GSE71989 to identify the difference in the mRNA level of REG3A, CD8A, IFN-*γ*, IL-12, and TGF-*β* between the tumors (*n*=115, *P*<0.01) and normal tissues (*n*=8, *P*<0.01). We next analyzed the correlation of REG3A and CD8A in the lymph node of PDAC under accession number GSE52171 (*n*=20, *P*<0.05) using the SPSS 20.0 software (Chicago, IL, USA). The GSE74262 and GSE75838 were downloaded to identify the potential target gene of STAT3 in maturation of DCs. Gene Ontology function were conducted for these genes with DAVID online analyses.

### Statistical analysis

Statistical analysis were performed by using a SPSS 11.0 software program (SPSS Software Products, Chicago, IL, USA). All data in this study were expressed as mean±S.D. Expression of proteins on western blot was quantified using the ImageJ analysis, which was also used in immunohistochemical quantitative analysis. Differences between multiple groups were examined for statistical significance using one-way analysis of variance (ANOVA). Differences between two groups were examined for statistical significance using the Student’s *t*-test. A *P*-value <0.01 denoted a statistically significant difference.

## Publisher’s Note:

Springer Nature remains neutral with regard to jurisdictional claims in published maps and institutional affiliations.

## References

[bib1] Grage-Griebenow E, Schafer H, Sebens S. The fatal alliance of cancer and T cells. How pancreatic tumor cells gather immunosuppressive T cells. Oncoimmunology 2014; 3: e29382. 10.4161/onci.29382 PMC4126073 25114835

[bib2] Huang B, Pan PY, Li Q, Sato AI, Levy DE, Bromberg J et al. Gr-1+CD115+ immature myeloid suppressor cells mediate the development of tumor-induced T regulatory cells and T-cell anergy in tumor-bearing host. Cancer Res 2006; 66: 1123–1131. 16424049 10.1158/0008-5472.CAN-05-1299

[bib3] Goebel L, Grage-Griebenow E, Gorys A, Helm O, Genrich G, Lenk L et al. CD4+ T cells potently induce epithelial–mesenchymal-transition in premalignant and malignant pancreatic ductal epithelial cells—novel implications of CD4+ T cells in pancreatic cancer development. Oncoimmunology 2015; 4: e1000083. 26137395 10.1080/2162402X.2014.1000083 PMC4485733

[bib4] Banchereau J, Steinman RM. Dendritic cells and the control of immunity. Nature 1998; 392: 245–252. 9521319 10.1038/32588

[bib5] Byrne WL, Mills KH, Lederer JA, O'Sullivan GC. Targeting regulatory T cells in cancer. Cancer Res 2011; 71: 6915–6920. 22068034 10.1158/0008-5472.CAN-11-1156 PMC4287207

[bib6] Liu X, Pu Y, Cron K, Deng L, Kline J, Frazier WA et al. CD47 blockade triggers T cell-mediated destruction of immunogenic tumors. Nat Med 2015; 21: 1209–1215. 26322579 10.1038/nm.3931 PMC4598283

[bib7] Liu X, Wang J, Wang H, Yin G, Liu Y, Lei X et al. REG3A accelerates pancreatic cancer cell growth under IL-6-associated inflammatory condition: Involvement of a REG3A-JAK2/STAT3 positive feedback loop. Cancer Lett 2015; 362: 45–60. 25779676 10.1016/j.canlet.2015.03.014

[bib8] Yin G, Du J, Cao H, Liu X, Xu Q, Xiang M. Reg3g promotes pancreatic carcinogenesis in a murine model of chronic pancreatitis. Dig Dis Sci 2015; 60: 3656–3668. 26182900 10.1007/s10620-015-3787-5

[bib9] Narushima Y, Unno M, Nakagawara K, Mori M, Miyashita H, Suzuki Y et al. Structure, chromosomal localization and expression of mouse genes encoding type III Reg, RegIII alpha, RegIII beta, RegIII gamma. Gene 1997; 185: 159–168. 9055810 10.1016/s0378-1119(96)00589-6

[bib10] Loncle C, Bonjoch L, Folch-Puy E, Lopez-Millan MB, Lac S, Molejon MI et al. IL17 functions through the novel REG3beta-JAK2-STAT3 inflammatory pathway to promote the transition from chronic pancreatitis to pancreatic cancer. Cancer Res 2015; 75: 4852–4862. 26404002 10.1158/0008-5472.CAN-15-0896 PMC4651828

[bib11] Zheng HC, Sugawara A, Okamoto H, Takasawa S, Takahashi H, Masuda S et al. Expression profile of the REG gene family in colorectal carcinoma. J Histochem Cytochem 2011; 59: 106–115. 21339177 10.1369/jhc.2010.956961 PMC3201118

[bib12] Gironella M, Calvo C, Fernandez A, Closa D, Iovanna JL, Rosello-Catafau J et al. Reg3beta deficiency impairs pancreatic tumor growth by skewing macrophage polarization. Cancer Res 2013; 73: 5682–5694. 23867474 10.1158/0008-5472.CAN-12-3057

[bib13] Cheng F, Wang HW, Cuenca A, Huang M, Ghansah T, Brayer J et al. A critical role for Stat3 signaling in immune tolerance. Immunity 2003; 19: 425–436. 14499117 10.1016/s1074-7613(03)00232-2

[bib14] Xia F, Cao H, Du J, Liu X, Liu Y, Xiang M. Reg3g overexpression promotes beta cell regeneration and induces immune tolerance in nonobese-diabetic mouse model. J Leukocyte Biol 2016; 99: 1131–1140. 26667474 10.1189/jlb.3A0815-371RRR

[bib15] Sun Y, Zhao Y, Wang X, Zhao L, Li W, Ding Y et al. Wogonoside prevents colitis-associated colorectal carcinogenesis and colon cancer progression in inflammation-related microenvironment via inhibiting NF-kappaB activation through PI3K/Akt pathway. Oncotarget 2016; 7: 34300–34315. 27102438 10.18632/oncotarget.8815 PMC5085157

[bib16] Xu Q, Fu R, Yin G, Liu X, Liu Y, Xiang M. Microarray-based gene expression profiling reveals genes and pathways involved in the oncogenic function of REG3A on pancreatic cancer cells. Gene 2016; 578: 263–273. 26719042 10.1016/j.gene.2015.12.039

[bib17] Dunn GP, Old LJ, Schreiber RD. The immunobiology of cancer immunosurveillance and immunoediting. Immunity 2004; 21: 137–148. 15308095 10.1016/j.immuni.2004.07.017

[bib18] Zhu S, Xu X, Liu K, Gu Q, Yang X. PAPep, a small peptide derived from human pancreatitis-associated protein, attenuates corneal inflammation *in vivo* and *in vitro* through the IKKalpha/beta/IkappaBalpha/NF-kappaB signaling pathway. Pharmacol Res 2015; 102: 113–122. 26388492 10.1016/j.phrs.2015.09.013

[bib19] Lim SO, Li CW, Xia W, Lee HH, Chang SS, Shen J et al. EGFR signaling enhances aerobic glycolysis in triple-negative breast cancer cells to promote tumor growth and immune escape. Cancer Res 2016; 76: 1284–1296. 26759242 10.1158/0008-5472.CAN-15-2478 PMC4775355

[bib20] van der Burg SH, Arens R, Ossendorp F, van Hall T, Melief CJ. Vaccines for established cancer: overcoming the challenges posed by immune evasion. Nat Rev Cancer 2016; 16: 219–233. 26965076 10.1038/nrc.2016.16

[bib21] Takeuchi S, Baghdadi M, Tsuchikawa T, Wada H, Nakamura T, Abe H et al. Chemotherapy-derived inflammatory responses accelerate the formation of immunosuppressive myeloid cells in the tissue microenvironment of human pancreatic cancer. Cancer Res 2015; 75: 2629–2640. 25952647 10.1158/0008-5472.CAN-14-2921

[bib22] Hirooka Y, Itoh A, Kawashima H, Hara K, Nonogaki K, Kasugai T et al. A combination therapy of gemcitabine with immunotherapy for patients with inoperable locally advanced pancreatic cancer. Pancreas 2009; 38: e69–e74. 19276867 10.1097/MPA.0b013e318197a9e3

[bib23] Ma Y, Adjemian S, Mattarollo SR, Yamazaki T, Aymeric L, Yang H et al. Anticancer chemotherapy-induced intratumoral recruitment and differentiation of antigen-presenting cells. Immunity 2013; 38: 729–741. 23562161 10.1016/j.immuni.2013.03.003

[bib24] Paterson Y. Rational approaches to immune regulation. Immunol Res 2003; 27: 451–462. 12857988 10.1385/IR:27:2-3:451

[bib25] Gutcher I, Becher B. APC-derived cytokines and T cell polarization in autoimmune inflammation. J Clin Invest 2007; 117: 1119–1127. 17476341 10.1172/JCI31720 PMC1857272

[bib26] Huggins A, Paschalidis N, Flower RJ, Perretti M, D'Acquisto F. Annexin-1-deficient dendritic cells acquire a mature phenotype during differentiation. FASEB J 2009; 23: 985–996. 19029200 10.1096/fj.08-119040

[bib27] Matsushita H, Vesely MD, Koboldt DC, Rickert CG, Uppaluri R, Magrini VJ et al. Cancer exome analysis reveals a T-cell-dependent mechanism of cancer immunoediting. Nature 2012; 482: 400–404. 22318521 10.1038/nature10755 PMC3874809

[bib28] Tseng D, Volkmer JP, Willingham SB, Contreras-Trujillo H, Fathman JW, Fernhoff NB et al. Anti-CD47 antibody-mediated phagocytosis of cancer by macrophages primes an effective antitumor T-cell response. Proc Natl Acad Sci USA 2013; 110: 11103–11108. 23690610 10.1073/pnas.1305569110 PMC3703977

[bib29] Figdor CG, de Vries IJ, Lesterhuis WJ, Melief CJ. Dendritic cell immunotherapy: mapping the way. Nat Med 2004; 10: 475–480. 15122249 10.1038/nm1039

[bib30] Geng L, Huang D, Liu J, Qian Y, Deng J, Li D et al. B7-H1 up-regulated expression in human pancreatic carcinoma tissue associates with tumor progression. J Cancer Res Clin Oncol 2008; 134: 1021–1027. 18347814 10.1007/s00432-008-0364-8 PMC12160751

[bib31] Pinzon-Charry A, Maxwell T, Lopez JA. Dendritic cell dysfunction in cancer: a mechanism for immunosuppression. Immunol Cell Biol 2005; 83: 451–461. 16174093 10.1111/j.1440-1711.2005.01371.x

[bib32] Liu J, Wang H, Yu Q, Zheng S, Jiang Y, Liu Y et al. Aberrant frequency of IL-10-producing B cells and its association with Treg and MDSC cells in non small cell lung carcinoma patients. Hum Immunol 2016; 77: 84–89. 26527508 10.1016/j.humimm.2015.10.015

[bib33] Tanikawa T, Wilke CM, Kryczek I, Chen GY, Kao J, Nunez G et al. Interleukin-10 ablation promotes tumor development, growth, and metastasis. Cancer Res 2012; 72: 420–429. 22123924 10.1158/0008-5472.CAN-10-4627 PMC3261323

[bib34] Zavasnik-Bergant T, Bergant Marusic M. Exogenous thyropin from p41 invariant chain diminishes cysteine protease activity and affects IL-12 secretion during maturation of human dendritic cells. PLoS ONE 2016; 11: e0150815. 26960148 10.1371/journal.pone.0150815 PMC4784741

[bib35] Tan MC, Goedegebuure PS, Belt BA, Flaherty B, Sankpal N, Gillanders WE et al. Disruption of CCR5-dependent homing of regulatory T cells inhibits tumor growth in a murine model of pancreatic cancer. J Immunol (Baltimore, Md : 1950) 2009; 182: 1746–1755. 10.4049/jimmunol.182.3.1746 PMC3738070 19155524

[bib36] Chauveau C, Remy S, Royer PJ, Hill M, Tanguy-Royer S, Hubert FX et al. Heme oxygenase-1 expression inhibits dendritic cell maturation and proinflammatory function but conserves IL-10 expression. Blood 2005; 106: 1694–1702. 15920011 10.1182/blood-2005-02-0494

[bib37] Tron K, Samoylenko A, Musikowski G, Kobe F, Immenschuh S, Schaper F et al. Regulation of rat heme oxygenase-1 expression by interleukin-6 via the Jak/STAT pathway in hepatocytes. J Hepatol 2006; 45: 72–80. 16510205 10.1016/j.jhep.2005.12.019

[bib38] Yasukawa H, Ohishi M, Mori H, Murakami M, Chinen T, Aki D et al. IL-6 induces an anti-inflammatory response in the absence of SOCS3 in macrophages. Nat Immunol 2003; 4: 551–556. 12754507 10.1038/ni938

[bib39] Bharadwaj U, Li M, Zhang R, Chen C, Yao Q. Elevated interleukin-6 and G-CSF in human pancreatic cancer cell conditioned medium suppress dendritic cell differentiation and activation. Cancer Res 2007; 67: 5479–5488. 17545630 10.1158/0008-5472.CAN-06-3963

[bib40] Willson TA, Jurickova I, Collins M, Denson LA. Deletion of intestinal epithelial cell STAT3 promotes T-lymphocyte STAT3 activation and chronic colitis following acute dextran sodium sulfate injury in mice. Inflamm Bowel Dis 2013; 19: 512–525. 23429443 10.1097/MIB.0b013e31828028ad PMC4330009

[bib41] Lutz MB, Kukutsch N, Ogilvie AL, Rossner S, Koch F, Romani N et al. An advanced culture method for generating large quantities of highly pure dendritic cells from mouse bone marrow. J Immunol Methods 1999; 223: 77–92. 10037236 10.1016/s0022-1759(98)00204-x

