# Supplementary material for: Acceleration of pancreatic tumorigenesis under immunosuppressive microenvironment induced by Reg3g overexpression
Source: Cell Death Dis. 2017 Sep 7;8(9):e3033–. doi: 10.1038/cddis.2017.424 (PMC5636971; doi:10.1038/cddis.2017.424)
Supplement: Supplementary Material [file cddis2017424x1.pdf]

SUPPLEMENTARY MATERIAL

Acceleration of pancreatic tumorigenesis under immunosuppressive microenvironment induced by Reg3g overexpression

Xiulan Liu[1,*], Zhongshi Zhou[1,*], Qi Cheng[1], Hongjie Wang[2], Hui Cao[1], Qianqian Xu[1], Yali Tuo[1], Li Jiang[3], You Zou[4], Hongyu Ren[5,*] and Ming Xiang[1,*]

[1]Department of Pharmacology, School of Pharmacy, Tongji Medical College, Huazhong University of Science and Technology, Wuhan 430000, China

[2]Section of Neurobiology, Torrey Pines Institute for Molecular Studies, Port Saint Lucie, Florida, USA

[3]Department of Biliary and Pancreatic Surgery, [4]Department of Gastrointestinal Surgery; Affiliated Tongji Hospital, Tongji Medical College, Huazhong University of Science and Technology, Wuhan 430000, China

[5]Department of Digestive Disease, Affiliated Xiehe Hospital, Tongji Medical College, Huazhong University of Science and Technology, Wuhan 430022, China

***Corresponding author**: Ming Xiang, Department of Pharmacology, School of Pharmacy, Tongji Medical College, Huazhong University of Science and Technology, Hangkong Road 13, Wuhan 430000, China, Tel: 0086-27-8369-2745; E-mail: xiangming@mails.tjmu.edu.cn; Hongyu Ren, Department of Digestive Disease, Affiliated Xiehe Hospital, Tongji Medical College, Huazhong University of Science and Technology, Liberation Avenue 1227, Wuhan 430022, China, Tel: 0086-27-8365-7879; E-mail: hongyur@hotmail.com.

*These authors contributed equally to the work.

Supplementary data

Table S1. The sequences of qRT-PCR primers

Genes	Forward (5’-----3’)	Reverse (5’-----3’)
β-actin	GATTACTGCTCTGGCTCCTAGC	GACTCATCGTACTCCTGCTTGC
IFN-γ	CATTCATGAGTATTGCCAAGTTTG	GCTGGATTCCGGCAACAG
TGF-β	AGCAACATGTGGAACTCTACC	GTATTCCGTCTCCTTGGTTCAG
IL-10	GGTTGCCAAGCCTTATCGGA	ACCTGCTCCACTGCCTTATCGGA
IL-12	AGGTGTCTTAGCCAGTCCCGAAACC	CTGAAGGCGTGAAGCAGGATGCAGA

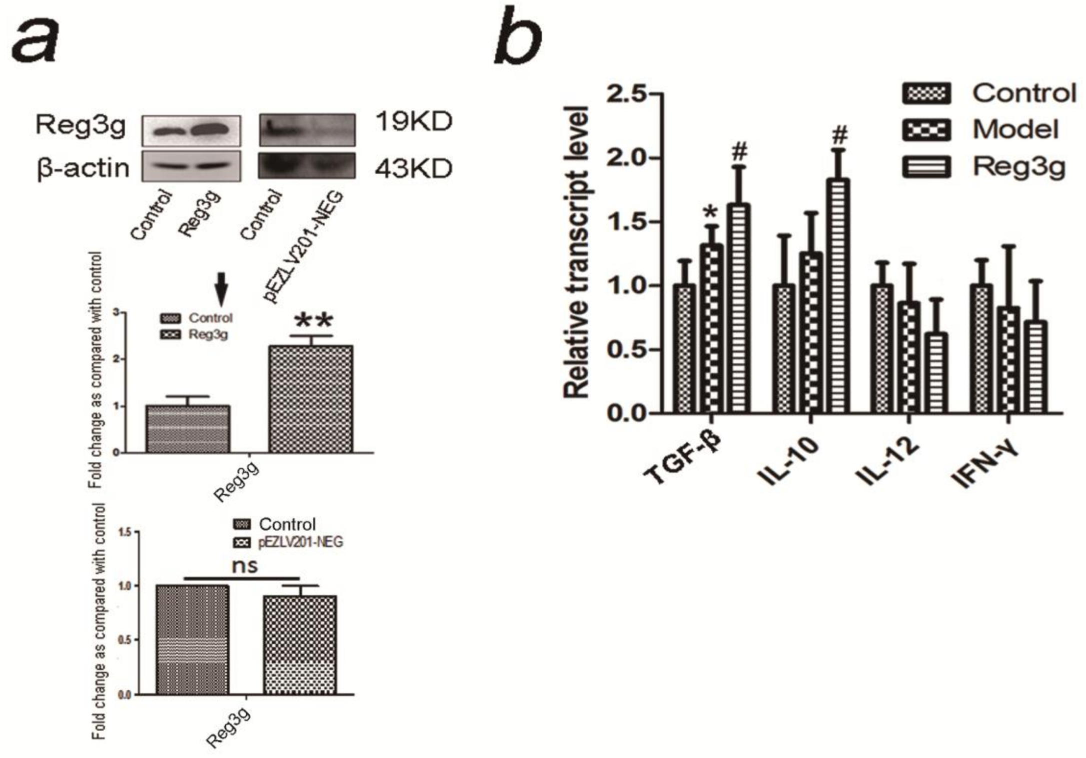

Supplementary Figure S1. (*a*) Expression of Reg3g were detected by western blot in Panc02 cells transfected with pReg3g lentiviral particles (Reg3g) or null letiviral vector (pEZLV201-NEG) or not (Control). (*b*) The mRNA expression of IL-10, IFN-γ, TGF-β and IL-12 in tumors was analyzed by qRT-PCR.

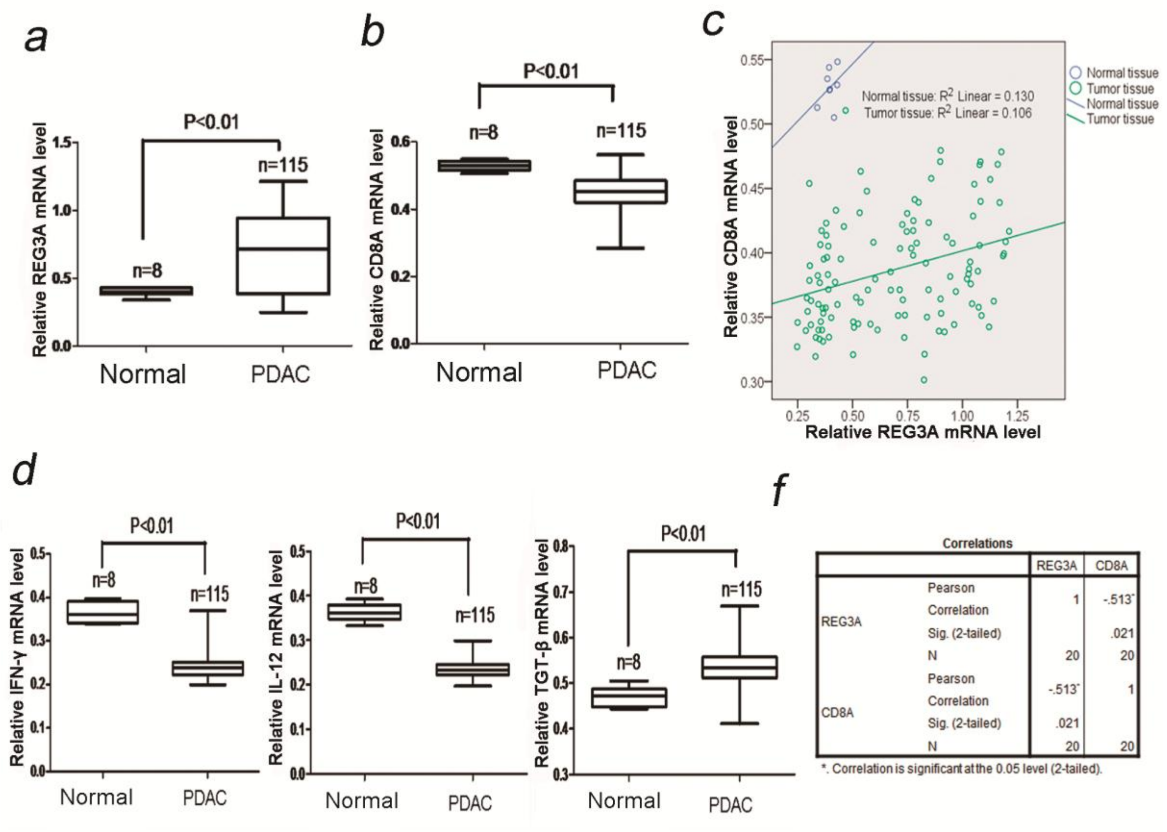

		REG3A	CD8A
REG3A	Pearson Correlation	1	-.513*
	Sig. (2-tailed)		.021
	N	20	20
CD8A	Pearson Correlation	-.513*	1
	Sig. (2-tailed)	.021	
	N	20	20

*. Correlation is significant at the 0.05 level (2-tailed).

Supplementary Figure S2. REG3A overexpression and lower level of CD8A in PDAC. (*a*, *b*) Expression profiles of REG3A and CD8A obtained from GEO datebase in normal tissues (n=8) and PDAC specimens (n=115). (*c*) The correlation between REG3A and CD8A expression in human normal tissues and PDAC specimens. (*d*) Statistical analysis of IFN-γ, IL-10 and TGF-β mRNA expression in normal tissues (n=8) and PDAC specimens (n=115). （*f*）The correlation between REG3A and CD8A expression in lymph node of PDAC patient (n=20).

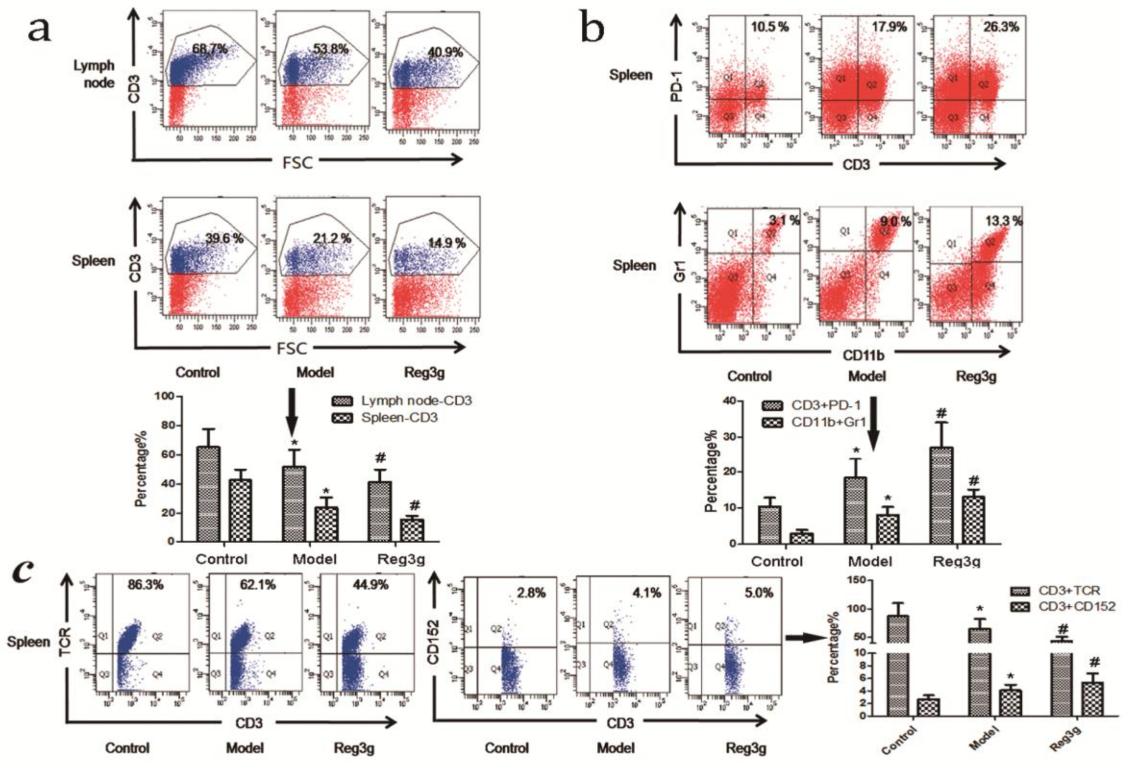

Supplementary Figure S3. Reg3g increased the expression of immunosuppressive molecules. (*a*) Percentage of CD3$^+$T cells in spleen and lymph mode. (*b*) The proportion of CD3$^+$PD-1$^+$ and MDSCs (CD11b$^+$Gr-1$^+$) in spleen. All the cells of (*a*) and (*b*) were spleen cells gating. (*c*) The proportion of TCR, CD152 accounting for CD3$^+$ T cells in spleen, all of the cells were in CD3$^+$T cells gating.

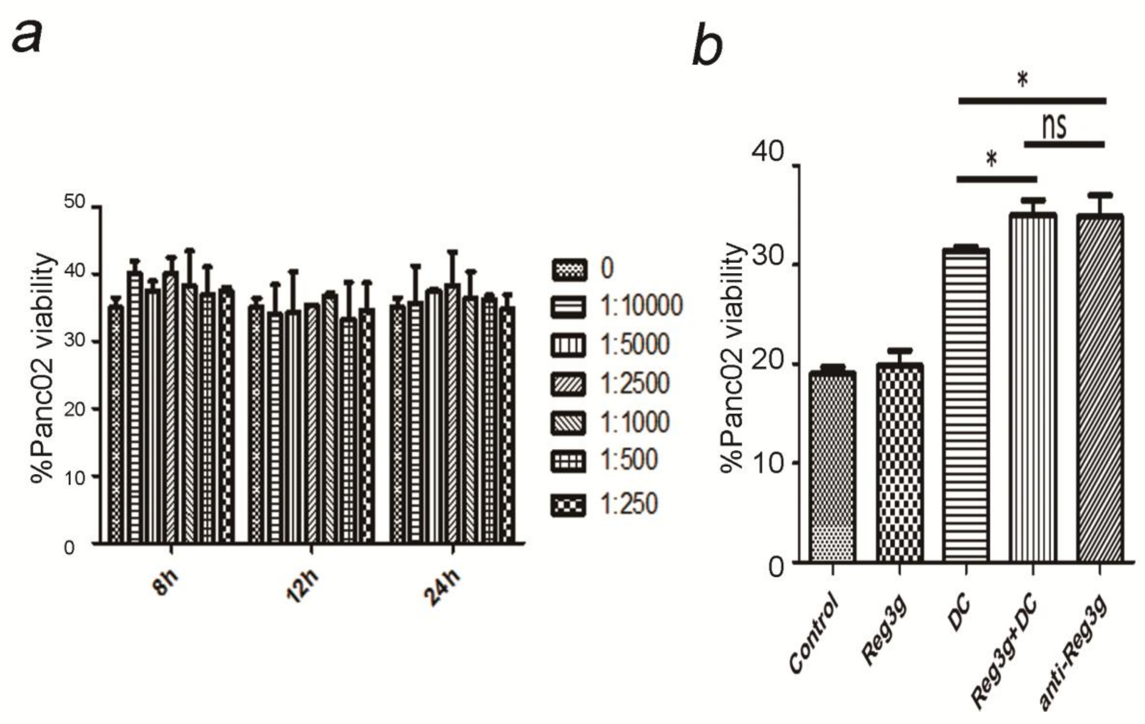

Supplementary Figure S4. Reg3g protein was neutralized by anti-Reg3g in conditioned media from DCs of TBM. (*a*) The Panc02 cell's viability was assessed by crystal violet staining after Panc02 cells incubated with 10% conditional media neutralized by anti-Reg3g. The ratio of anti-Reg3g in conditioned media was 1:10000, 1:5000, 1:2500, 1:1000, 1:500, 1:250 for 8h, 12h, 24h, "0" represented no anti-Reg3g. (*b*). The DC-mediated effects on the Panc02 cell's viability was confirmed through Panc02 cell incubation with conditioned media neutralized by anti-Reg3g. Control: media alone; Reg3g: 100ng/ml Reg3g; DC: conditioned media from DCs of TBM; Reg3g+DC: conditioned media from DCs of TBM in combination with 100ng/ml Reg3g; anti-Reg3g: neutralized conditioned media from DCs of TBM in combination with 100ng/ml Reg3g by anti-Reg3g at the ratio of 1:1000 for 24h.

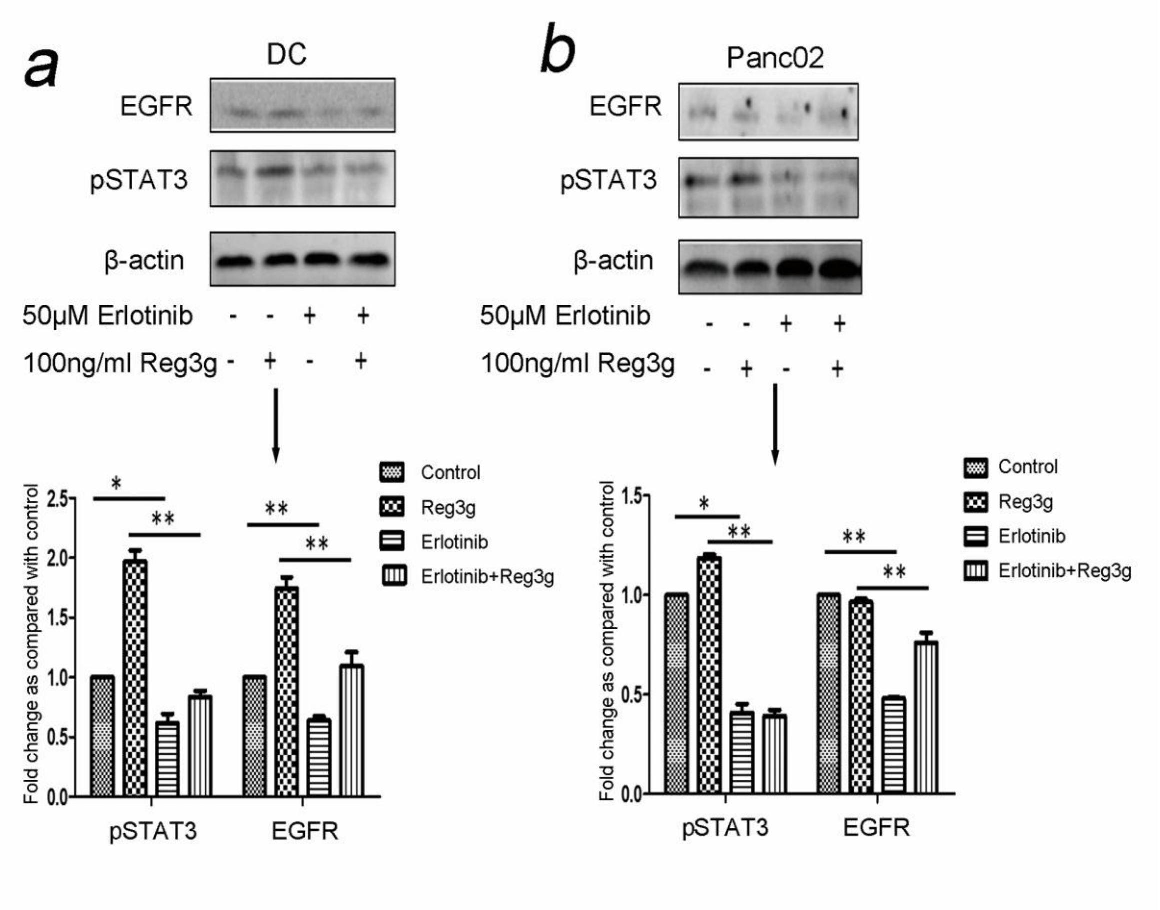

Supplementary Figure S5. EGFR mediated STAT3 signaling activation in DCs and Panc02 cells by Reg3g. (a) The expression of EGFR and pSTAT3 in DCs by EGFR inhibitor. (*b*) The expression of EGFR and pSTAT3 in Panc02 cells by EGFR inhibitor.
